# Supplementary material for: Fisheries portfolio diversification and turnover buffer Alaskan fishing communities from abrupt resource and market changes
Source: Nat Commun. 2017 Jan 16;8:14042. doi: 10.1038/ncomms14042 (PMC5241831; doi:10.1038/ncomms14042)
Supplement: Supplementary Information — Supplementary Figures and Supplementary Tables [file ncomms14042-s1.pdf]

Supplementary Material

Supplementary Table 1: Model Selection for dynamic factor analysis models evaluating common patterns in time series of commercial fisheries catches in 60 stocks around Alaska

Stocks are delineated by species or species group and area harvested. We tested up to two trend models. We considered two error structures; where all stocks share a single observation error (DE), where all stocks have individual observation error (DUE). A one-trend model indicates the most commonly shared trend among the 60 time series of commercial fisheries catches.

Number of trends	Error structure	Num. parameters	AIC	ΔAIC
**1**	**DE**	**61**	**5643**	**0**
1	DUE	120	5732	89
2	DE	120	5678	35
2	DUE	179	5720	77

Supplementary Table 2: Model Selection for dynamic factor analysis models evaluating common patterns in time series of commercial fisheries revenue from 105 Alaskan communities We tested up to two trend models. We considered two error structures; where all stocks share a single observation error (DE), where all stocks have individual observation error (DUE). A two-trend model indicates the two most commonly shared trends among the 105 time series of commercial fisheries revenue.

Number of trends	Error structure	Num. parameters	AIC	ΔAIC
1	DE	106	6972	1316
1	DUE	210	6361	884
2	DE	210	6257	599
**2**	**DUE**	**314**	**5658**	**0**

Supplementary Table 3: Regression results explaining changes in community revenues against changes in catch and changes in price Changes in revenue are measured as the percent change in revenue from commercial fishing for each community from before (1980-1989) and after (1990-1999) the regime shifts. Changes in catch are measured as the percent change in catch (pounds harvested) from commercial fishing for these same years. Percent changes in price are computed from prices weighted by the proportion of commercial harvest by weight. To assess relative strength of covariates, effect sizes were computed using a separate regression of z-score standardized variables. Best model includes full main effects and interaction terms. The full model explains 93% of the variation in in change in revenue (R^2=0.9268, $p < 0.001$).

Covariate	Coefficient	Std. Error	P-value	Effect Size
Intercept	-6.790	2.295	<0.0001	
Change in catch	0.913	0.040	<0.0001	1.19
Change in price	0.801	0.060	<0.0001	0.84
Interaction between change in catch and change in price	0.008	0.001	<0.0001	0.43

Supplementary Table 4: Model selection for regression analysis of changes in revenue in response to different types of diversification We tested richness (the number of fisheries) and diversity (a combination of richness and evenness as measured by the Reciprocal Simpson's Index). Both variables are $\log_{10}$ transformed. To assess relative strength of covariates, effect sizes were computed using a separate regression of z-score standardized variables.

Model Covariates	Number of parameters	AIC	ΔAIC	Effect Size
Number of fisheries (richness)	2	735.9	6.0	9.9
**Diversity**	**2**	**729.9**	**0**	**13.7**

Supplementary Table 5: Regression model selection for changes in revenue as a function of diversification and turnover Changes in revenue are measured as the percent change in revenue from commercial fishing for each community from before (1980-1989) and after (1990-1999) the regime shifts. Diversification is measured as Reciprocal Simpson's Diversity ($\log_{10}$) and turnover is measured using Jaccard's Dissimilarity index ($\log_{10}$).

Model covariates	Number of parameters	AIC	ΔAIC
Diversification	2	1001.7	5.66
Turnover	2	1001.6	5.65
Diversification + Turnover	3	999.58	3.54
Diversification + Diversification*Turnover	**3**	**996.04**	**0**
Diversification + Turnover + Diversification*Turnover	4	997.66	1.62

Supplementary Table 6: Regression parameters for the best linear model fit for changes in community commercial fishing revenues as a function of diversification and turnover

Changes in revenue are measured as the percent change in revenue from commercial fishing for each community from before (1980-1989) and after (1990-1999) the regime shifts. Diversification is measured as Reciprocal Simpson's Diversity (log_{10}) and turnover is measured using Jaccard's Dissimilarity index (log_{10}). To assess relative strength of covariates, effect sizes were computed using a separate regression of z-score standardized variables. The best model includes main effects on diversification and interaction terms. The full model explains 26% of the variation in in change in revenue (R^2=0.26, p < 0.00001).

Covariate	Coefficient	Std. error	P-value	Effect size
Intercept	-41.43	3.965	<0.0001	
Diversification	123.5	21.78	<0.0001	0.307
Diversification*Turnover	71.52	25.81	<0.01	0.187

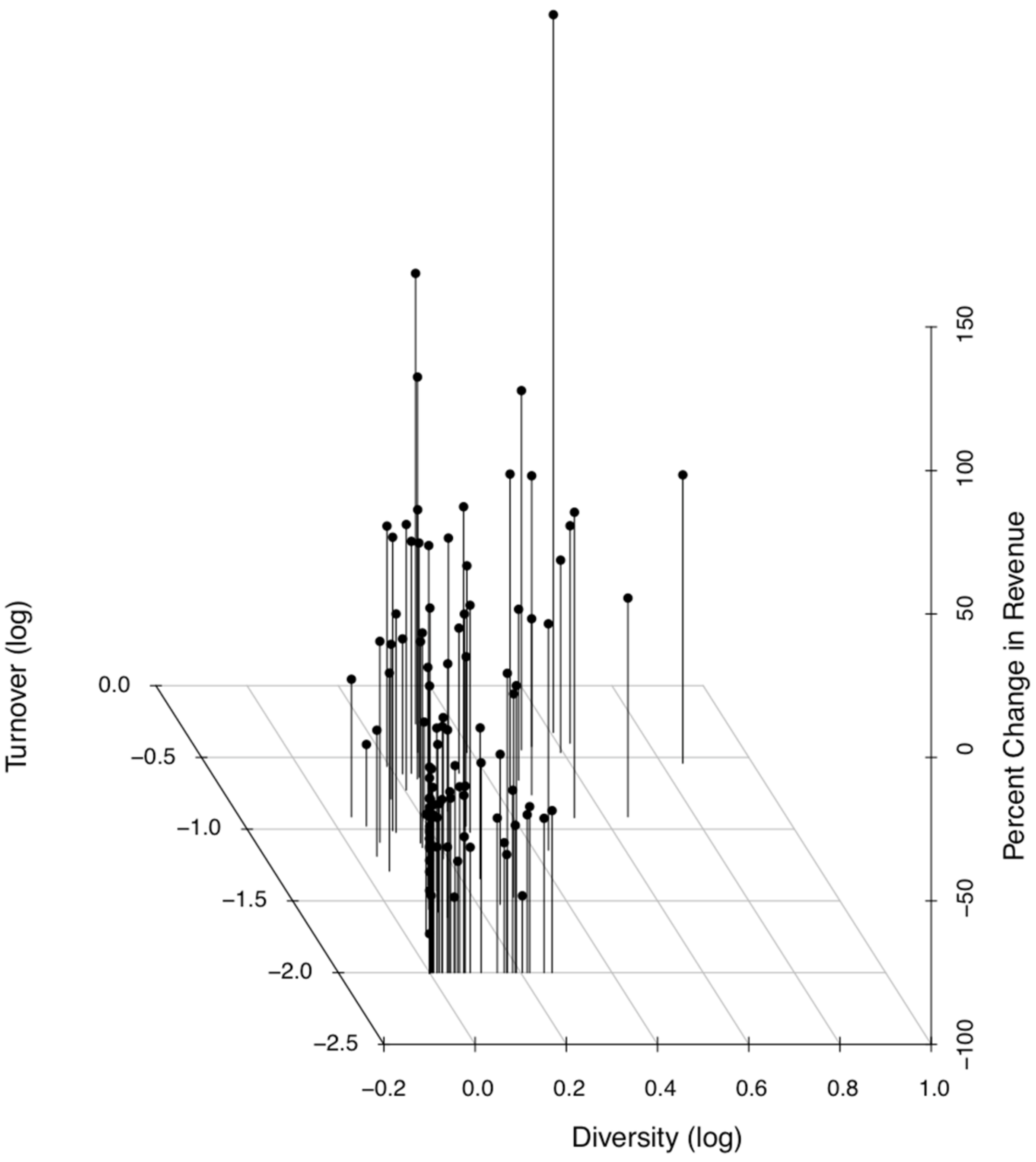

Supplementary Figure 1: Diversification and turnover of fishing opportunities buffer against abrupt shifts Observed changes in revenues from before (1980-1989) and after (1990-1999) are plotted against diversity and turnover of fishing opportunities for 105 Alaskan fishing communities. Diversity, as measured by the reciprocal of Simpson diversity (log scale), indicates total portfolio size and distribution among component stocks (often referred to as alpha

diversity). Turnover (often referred to as beta diversity) is Jaccard's dissimilarity index measuring changes in the in the relative contribution of stocks to a communities overall portfolio (by catch). Regression analysis indicates the best linear model between changes in revenue as explained by Simpson's diversity and turnover includes a significant interaction (R^2=0.26, $p<0.0001$, Supplementary Table 5, Supplementary Table 6).
